# Sensing chemical-induced genotoxicity and oxidative stress via yeast-based reporter assays using NanoLuc luciferase

**DOI:** 10.1371/journal.pone.0294571

**Published:** 2023-11-22

**Authors:** Minami Shichinohe, Shun Ohkawa, Yuu Hirose, Toshihiko Eki

**Affiliations:** 1 Molecular Genetics Laboratory, Toyohashi, Japan; 2 Department of Applied Chemistry and Life Science, Laboratory of Genomics and Photobiology, Toyohashi University of Technology, Toyohashi, Aichi, Japan; CNR, ITALY

## Abstract

Mutagens and oxidative agents damage biomolecules, such as DNA; therefore, detecting genotoxic and oxidative chemicals is crucial for maintaining human health. To address this, we have developed several types of yeast-based reporter assays designed to detect DNA damage and oxidative stress. This study aimed to develop a novel yeast-based assay using a codon-optimized stable or unstable NanoLuc luciferase (*yNluc* and *yNluCP*) gene linked to a DNA damage- or oxidative stress-responsive promoter, enabling convenient sensing genotoxicity or oxidative stress, respectively. End-point luciferase assays using yeasts with a chromosomally integrated *RNR3* promoter (^P^*RNR3*)-driven *yNluc* gene exhibited high levels of chemiluminescence via NanoLuc luciferase and higher fold induction by hydroxyurea than a multi-copy plasmid-based assay. Additionally, the integrated reporter system detected genotoxicity caused by four different types of chemicals. Oxidants (hydrogen peroxide, *tert*-butyl hydroperoxide, and menadione) were successfully detected through transient expressions of luciferase activity in real-time luciferase assay using yeasts with a chromosomally integrated *TRX2* promoter (^P^*TRX2*)-linked *yNlucCP* gene. However, the luciferase activity was gradually induced in yeasts with a multi-copy reporter plasmid, and their expression profiles were notably distinct from those observed in chromosomally integrated yeasts. The responses of *yNlucCP* gene against three oxidative chemicals, but not diamide and zinc oxide suspension, were observed using chromosomally integrated reporter yeasts. Given that yeast cells with chromosomally integrated ^P^*RNR3*-linked *yNluc* and ^P^*TRX2*-linked *yNlucCP* genes express strong chemiluminescence signals and are easily maintained and handled without restrictive nutrient medium, these yeast strains with NanoLuc reporters may prove useful for screening potential genotoxic and oxidative chemicals.

## Introduction

DNA damage caused by mutagenic chemicals increases the risk of cancer by causing genetic mutations in humans [1]. Oxidative damage is constantly generated by cellular metabolism-derived reactive oxygen species (ROS) and/or environmental oxidative chemicals [2]. Endogenous ROS and exogenous oxidants damage cellular molecules, such as DNA, leading to cellular dysfunction and genetic mutations; additionally, they are implicated in carcinogenesis, neurodegenerative diseases, cardiovascular diseases, inflammation, and aging [3]. Bioassays have been developed to assess potential genotoxic and oxidative chemicals. To access potential genotoxicity in eukaryotes, *Saccharomyces cerevisiae*-based reporter assays have been developed using reporter genes linked to several DNA damage-inducible promoters, including *RNR2* [4,5], *RNR3* [5–9], *RAD54* [4,6,10], *RAD51* [11], *HUG1* [9,12], *PLM2*, and *DIN7* [13]. The transcriptional induction of these genes is governed by the activation of the DNA damage checkpoint pathway in response to DNA damage caused by mutagens [14]. The budding yeast *S*. *cerevisiae* is an eukaryote like humans that can be easily manipulated; therefore, yeast-based reporter assays are suitable for assessing the potential genotoxicity in mammals [15]. However, oxidative damage in animals has been evaluated by measuring the amounts of oxidized biomolecules, such as 8-hydroxy-2-deoxyguanosine, in urine and blood samples [16]. Yu et al. conducted a yeast-based assay for sensing oxidative stress using a redox-sensitive GFP [17]. Several oxidative stress-inducible genes, including *TRX2* and *CTT1* have been identified by genome-wide gene expression analyses in yeast [18–22]. However, only a few studies using yeast-based reporter assays with an oxidative stress-inducible reporter gene have been reported so far [23–26]. Dolz-Edo et al. [23] investigated the transcriptional expression of a destabilized luciferase reporter gene linked to four oxidative stress–responsive promoters (*GRE2*, *CTT1*, *SOD2*, and *CCP1*) in yeast strains following exposure to hydrogen peroxide and menadione. They observed concentration-dependent transient expressions induced by these oxidants. Lewinska et al. [25] and Jayaraman et al. [24] developed yeast reporter strains with *YHB1* and *TRX2* promoter-linked *gfp* genes and observed GFP expression caused by nitric oxide donors and two oxidants (hydrogen peroxide and diamide), respectively.

Previously, we developed a novel yeast-based assay system using both a sensor and an *Escherichia coli lacZ*-reporter plasmid, which could detect low concentrations of genotoxic agents more efficiently than conventional reporter systems [5]. In addition, yeast-based assays for genotoxicity using reporter plasmids carrying the *Cypridina noctiluca* secretory luciferase [8], *GFP*, and firefly luciferase [26] genes linked to the ribonucleotide reductase subunit 3 gene promoter (^P^*RNR3*) have been successfully used to detect chemical genotoxicity. In addition, we developed a novel yeast-based assay for detecting oxidative agents using a reporter gene encoding unstable firefly luciferase containing the CL1 and proline-glutamate-serine-threonine-rich (PEST) protein destabilizing sequence, linked to the thioredoxin 2 gene promoter (^P^*TRX2*) [26]. Many oxidative stress-responsive genes are regulated by transcription factors, such as Yap1p [27], and transiently expressed during 100 min after oxidant treatment [23]. Therefore, the system used in our aforementioned study was based on detecting the transient expression of luciferase caused by oxidative agents using the real-time luciferase assay with yeasts and a multi-copy plasmid carrying a ^P^*TRX2*-linked destabilized luciferase gene.

However, although genotoxicants and oxidative agents were detected using these plasmid-based reporter assays, one issue remained unresolved. A nutrient-selective medium was required to maintain the plasmids in the yeast during the culture and assay, making the assay time-consuming and inconvenient. In the present study, we generated yeast cells carrying a chromosomally integrated reporter gene using CRISPR/Cas9. The luminescence intensity in the chromosomal integration reporter assay is expected to decrease considerably due to the presence of a single copy of the reporter gene; therefore, a codon-optimized NanoLuc luciferase (yNluc) was used as a reporter to resolve this issue. NanoLuc luciferase is an engineered luciferase originating from the deep-sea shrimp *Oplophorus gracilirostris* [28]. It catalyzes the commercialized substrate Nano-Glo^®^ and produces a signal approximately 150-fold brighter than firefly luciferase in an ATP-independent manner [29]. Masser et al. first reported the development of a yeast-based reporter assay using yNluc and its usefulness [30]; this was followed by the development of a yeast-based estrogen sensor by Cevenini et al. using NanoLuc [31].

Thus, the current study aimed to compensate for the decreased luminescence in the assay using a chromosomally integrated reporter yeast by virtue of its strong luminescence.

## Materials and methods

### Chemicals

Methyl methanesulfonate (MMS), hydroxyurea (HU), mitomycin C (MMC), *tert*-butyl hydroperoxide (*t*-BHP), menadione, diamide, and zinc oxide were purchased from Sigma-Aldrich Inc. (St. Louis, MO). Camptothecin (CPT) and hydrogen peroxide (H_2_O_2_) were obtained from FUJIFILM Wako Pure Chemical Corp. (Osaka, Japan), and phleomycin (Phl) was purchased from InvivoGen (Hong Kong). CPT and menadione were dissolved in dimethyl sulfoxide (Sigma-Aldrich Inc.) and diluted with distilled water. Zinc oxide does not dissolve completely in water; therefore, a sonicated zinc oxide suspension (0.32 g/L) was serially diluted in distilled water.

### Strains

The *S*. *cerevisiae* strains prepared and used in this study are shown in Table 1. Yeast cells were grown at 30°C in yeast-peptone-dextrose (YPD) medium containing 1% yeast extract, 2% peptone, and 2% glucose [32]. Those with luciferase reporter plasmids were maintained and cultured in synthetic dextrose minimal (SD) medium without histidine (ForMedium, Hunstanton, UK).

**Table 1:** The yeast strains used in this study.

Strains	Genotype	Source
BY4741	*MAT***a**, *his3-Δ1*, *leu2-Δ0*, *met15-Δ0*, *ura3-Δ0*	Invitrogen
IMX672	*MAT***a**, *ura3-52*, *trp1-289*, *leu2-3*,*112*, *his3Δ*, *can1Δ*::*cas9-natNT2*	EUROSCRAF
BY4741-^P^*RNR3-yNluc*	*MAT***a**, *his3-Δ1*, *leu2-Δ0*, *met15-Δ0*, *ura3-Δ0*, *can1Δ*:: ^P^*RNR3-yNluc*	This study
BY4741-^P^*TRX2-yNlucCP*	*MAT***a**, *his3-Δ1*, *leu2-Δ0*, *met15-Δ0*, *ura3-Δ0*, *can1Δ*:: ^P^*TRX2-yNlucCP*	This study

### Preparations of yNluc reporter plasmids

Two types of yNluc reporter plasmids (pESC-HIS*ΔGAL1/10-*^P^*RNR3-yNluc* and pESC-HIS*ΔGAL1/10-*^P^*TRX2-yNlucCP*) derived from the multi-copy plasmid vector pESC-HIS (Agilent, Santa Clara, CA) were prepared to detect the genotoxicity and oxidative stress, respectively (Table 2). The *yNluc* and *yNlucCP* genes encode a codon-optimized Nluc and an unstable Nluc with the CL1 and PEST protein destabilizing sequence, respectively. Both DNAs were prepared by polymerase chain reaction (PCR) using the KOD FX Neo DNA polymerase (Toyobo, Tokyo), the primer sets (yNluc-5’-F and pESChis2DSma-yNlucend-IFR for *yNluc* gene, and yNluc-5’-F and pESChis2DSma-yNlucPEST-IFR for *yNlucCP* gene) (S1 Table), and the *yNluc*-pUC57-Amp plasmid DNA (Azenta Life Sciences, South Plainfield, NJ) containing a synthesized *yNlucCP* gene as a template (S1 Fig). The promoter fragments of *RNR3* and *TRX2* were amplified from yeast genomic DNA (BY4741) using KOD FX Neo, the primer set (pESChis2DSma-RNR3P-IFF and RNR3P-yNluc25-R for *RNR3* and pESChis2DSma-TRX2P500-IFF and TRX2P500-yNluc25-R for *TRX2*), and the promoter-containing plasmid DNA (pESC-HIS*ΔGAL1/10-*^P^*RNR3-luc2* and pESC-HIS*ΔGAL1/10-*^P^*TRX2-luc2CP* for *RNR3* and *TRX2*, respectively). ^P^*RNR3* and ^P^*TRX2* fragments were connected to *yNluc* and *yNlucCP* DNAs by PCR using the primer sets (pESChis2DSma-RNR3P-IFF and pESChis2DSma-yNlucend-IFR, and pESChis2DSma-TRX2P500-IFF and pESChis2DSma-yNlucPEST-IFR, respectively) and then cloned into the *Sma* I site of the pESC-HIS*ΔGAL1/10* plasmid using the In-Fusion HD cloning kit (Takara, Osaka). The resultant pESC-HIS*ΔGAL1/10-*^P^*RNR3-yNluc* and pESC-HIS*ΔGAL1/10-*^P^*TRX2-yNlucCP* were used for the genotoxicity and oxidative stress assays, respectively. The nucleotide sequences of the cloned DNAs, along with their flanking regions in the recombinant plasmids, were determined in both directions using a BigDye^®^ Terminator v3.1 Cycle Sequencing Kit (Thermo Fisher Scientific Inc., Waltham, MA) and an automated DNA sequencer (Applied Biosystems model 3130xl Genetic Analyzer) by Macrogen Japan (Tokyo, Japan). The sequence data were assembled and analyzed using the ATGC and Genetyx software (version 13, Genetyx Co., Tokyo). The oligo DNAs used in this study were synthesized by FASMAC (Atsugi, Japan) and Integrated DNA Technologies (Coralville, IO).

**Table 2:** The plasmids used in this study.

Plasmid	Description
pRS415-LEU-Cas9	A pRS415 plasmid containing *cas9* expression cassette DNA at *Sma* I site
pMEL10	An RNA expression plasmid with *URA3* marker and guide RNA (gRNA) for *CAN1* gene, which was generated by Mans et al. [33] and obtained from EUROSCARF
*yNluc*-pUC57-Amp	A pUC57 plasmid containing a codon-optimized *yNanolucCP* gene, which was synthesized by Azenta Life Sciences (GENEWIZ) based on the nucleotide sequence of pCA955 kindly provided by Prof. Andréasson (Stockholm University) [30]
pESC-HIS*ΔGAL1/10*	A *GAL1/10* promoter-removed derivative of the pESC-HIS vector [26]
pESC-HIS*ΔGAL1/10-*^P^*RNR3-luc2*	pESC-HIS*ΔGAL1/10* with a *RNR3* promoter-linked *luc2* gene [26]
pESC-HIS*ΔGAL1/10-*^P^*TRX2-luc2CP*	pESC-HIS*ΔGAL1/10* with a *TRX2* promoter-linked *luc2CP* gene [26]
pESC-HIS*ΔGAL1/10-*^P^*RNR3-yNluc*	A multi-copy yNluc reporter plasmid driven by *RNR3* promoter, prepared in this study
pESC-HIS*ΔGAL1/10-*^P^*TRX2-yNlucCP*	A multi-copy unstable yNlucCP reporter plasmid driven by *TRX2* promoter, prepared in this study

### Preparation of chromosomally integrated reporter strains

Two BY4741-derived yeast strains carrying a chromosomally integrated ^P^*RNR3-yNluc* and ^P^*TRX2-luc2CP* gene (BY4741-^P^*RNR3-yNluc* and BY4741-^P^*TRX2-yNlucCP*) were prepared by replacing *CAN1* with CRISPR/Cas9 (Table 1), as described by Mans et al. [33]. We used CRISPR/Cas9 for chromosomal integration of the reporter construct instead of classical genomic integration because of the high efficiency of integrations at the correct loci and the capability to perform multiple chromosomal integrations without any restrictions regarding the number of nutrient-selective genes. In brief, yeast cells with the Cas9-expression plasmid pRS415-LEU-Cas9 were co-transformed by the *CAN1*-gRNA expression plasmid pMEL10 and the promoter-linked reporter DNA with 5’- and 3’-flanking sequences of *CAN1* at each end. The resultant *LEU*^+^- and *HIS*^+^-transformants were investigated by colony PCR using allele-specific primer sets (S1 Table) with KOD FX Neo polymerase to confirm the integration of the promoter-linked reporter gene at the *CAN1* locus on chromosome V (S2 Table). The DNAs for integration were prepared by amplifying the ^P^*RNR3-yNluc* and ^P^*TRX2-luc2CP* DNAs from pESC-HIS*ΔGAL1/10-*^P^*RNR3-yNluc* and pESC-HIS*ΔGAL1/10-*^P^*TRX2-yNlucCP*, respectively, using KOD FX Neo with the primer set (TADH1-5F2-25CAN1-Rtail and TCYC1-3R3-25CAN1-Ftail). The 5’- and 3’-flanking DNAs were prepared by PCR using KOD FX Neo and the primer sets (5’-CAN1-F and 5-CAN1-R_5F2_30tail, and 3-CAN1-F_3R3_30tail and 3’-CAN1-R), respectively. Purified promoter-reporter DNA and 5’-flanking DNA were connected by PCR using the primer set (5’-CAN1-F and TCYC1-3R3-25CAN1-Ftail). The resultant DNAs were further connected with 3’-flanking DNA by PCR using the 5’-CAN1-F and 3’-CAN1-R primer set to generate the promoter-linked reporter DNA with the 5’- and 3’- *CAN1*flanking sequences.

### Yeast transformation

Yeast cells were transformed with pESC-HIS-derived reporter plasmids using a lithium acetate protocol modified by Gietz et al. [34]. The transformants were selected on SD agar plates without histidine (ForMedium), and independent colonies were streaked onto a fresh selection of agar plates before use. For gene integration, the yeast cells were first transformed by the Cas9-expression plasmid pRS415-LEU-Cas9 with the *LEU2* marker. Then, the *LEU*^+^-transformants were co-transformed by the *CAN1*-gRNA expression plasmid pMEL10 with the *HIS3* marker and a promoter-linked reporter gene fragment as described above. The integrated strains were selected on SD agar plates without leucine and histidine.

### End-point luciferase assay for genotoxicity

Yeast cells with pESC-HIS*ΔGAL1/10-*^P^*RNR3-yNluc* were grown on SD agar plates without histidine and cultured with 10 mL of histidine-free SD medium in a 50 mL conical tube at 30°C with continuous shaking for 24 h. Yeast cells with chromosomally integrated ^P^*RNR3-yNluc* gene (BY4741-^P^*RNR3-yNluc*) were cultured in 10 mL of the YPD medium. The cells were collected by centrifugation to remove the growth medium and suspended in YPD medium at an absorbance of 600 nm (OD_600_) of approximately 1.0. Subsequently, 100 μL of the yeast suspensions were placed in a 96-well white plate (Coster, No. 3912) in triplicates, and the tested chemicals were added at the indicated concentrations. The yeast cells in the microplate were incubated at 30°C for 24 h under saturated humidity in a Tupperware. Next, a 100 μL-aliquot of the diluted cell suspension was transferred into a new 96-well white plate containing 10 μL of 100-fold-diluted Nano-Glo^®^ Luciferase Assay Substrate (Promega, Madison, WI). After 30 min, the *A*_600_ and chemiluminescence intensity were measured using a multimode plate reader (Tecan Infinite M1000, Männedorf, Switzerland). The luciferase activity is shown in arbitrary units defined as the luminescence (counts) in 1 s normalized by the *A*_600_. The fold induction was calculated as the ratio of the luciferase activity in the presence and absence of each test chemical. In this study, we investigated the responses of a chromosomally integrated ^P^*RNR3-yNluc* gene following exposure to four different types of genotoxic chemicals: methyl methanesulfonate (MMS), an alkylating agent, Phyleomycin (Phl) that induces DNA double-strand breaks, mitomycin C (MMC) causing DNA strand cross-links, and camptothecin (CPT), a DNA topoisomerase I inhibitor that breaks replication forks.

### Real-time luciferase assay for oxidative stress

Yeast cells with pESC-HIS*ΔGAL1/10-*^P^*TRX2-luc2CP* and chromosomally integrated ^P^*TRX2-luc2CP* gene (BY4741-^P^*TRX2-luc2CP*) were pre-cultured and cultured in a microtiter plate without chemicals as described in the previous section. A 100 μL-aliquot of the diluted cell suspension was transferred into a new 96-well white plate containing 10 μL of 100-fold-diluted Nano-Glo^®^ Luciferase Assay Substrate, and oxidative chemicals were quickly added at the indicated concentrations. At time zero, the *A*_600_ and chemiluminescence intensity were measured in a multimode plate reader, and the luminescence intensity was subsequently determined every 10 min until 1 h and 20 min at 28°C. The normalized luciferase activity is shown in arbitrary units, as described in the previous section. The relative maximal activity was defined as the percentage of the highest peak luciferase activity at each concentration divided by the highest peak activity among all the concentrations tested. The results were plotted against the concentrations of the oxidative agents.

### Statistical analysis

A two-tailed paired Student’s *t*-test using the T.TEST function in Microsoft Excel was employed for the statistical analysis. A *p*-value of less than 0.01 was considered statistically significant.

## Results

### Detection of genotoxicity using yeast strains carrying an *RNR3* promoter-linked *yNluc* gene on a multi-copy plasmid and a chromosome

**Fig 1:**
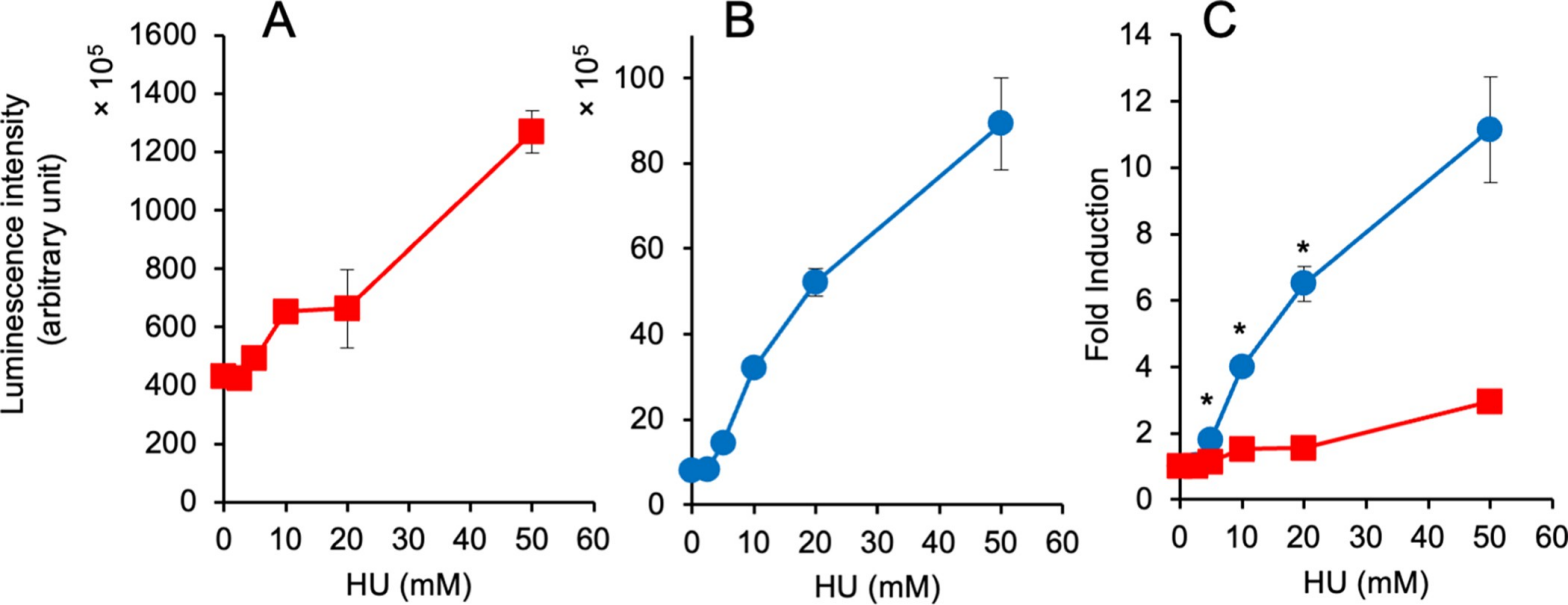
Response of ^P^*RNR3*-linked *yNluc* gene in two reporter strains after hydroxyurea (HU) treatment. The luciferase-derived luminescence in yeast cells carrying a multi-copy reporter plasmid (**A**) and a chromosomally integrated reporter gene (**B**) were measured after exposure to various concentrations of HU for 24 h. The luminescence intensities were normalized using the *A*_600_ value and plotted against the HU concentrations with the standard deviations. The fold inductions in two yeast strains are shown in (**C**). * Statistically significant (*p* < 0.01, Student’s *t*-test). The raw dataset for Fig 1 and statistical analysis are shown in S3 and S4 Tables, respectively.

**Fig 2:**
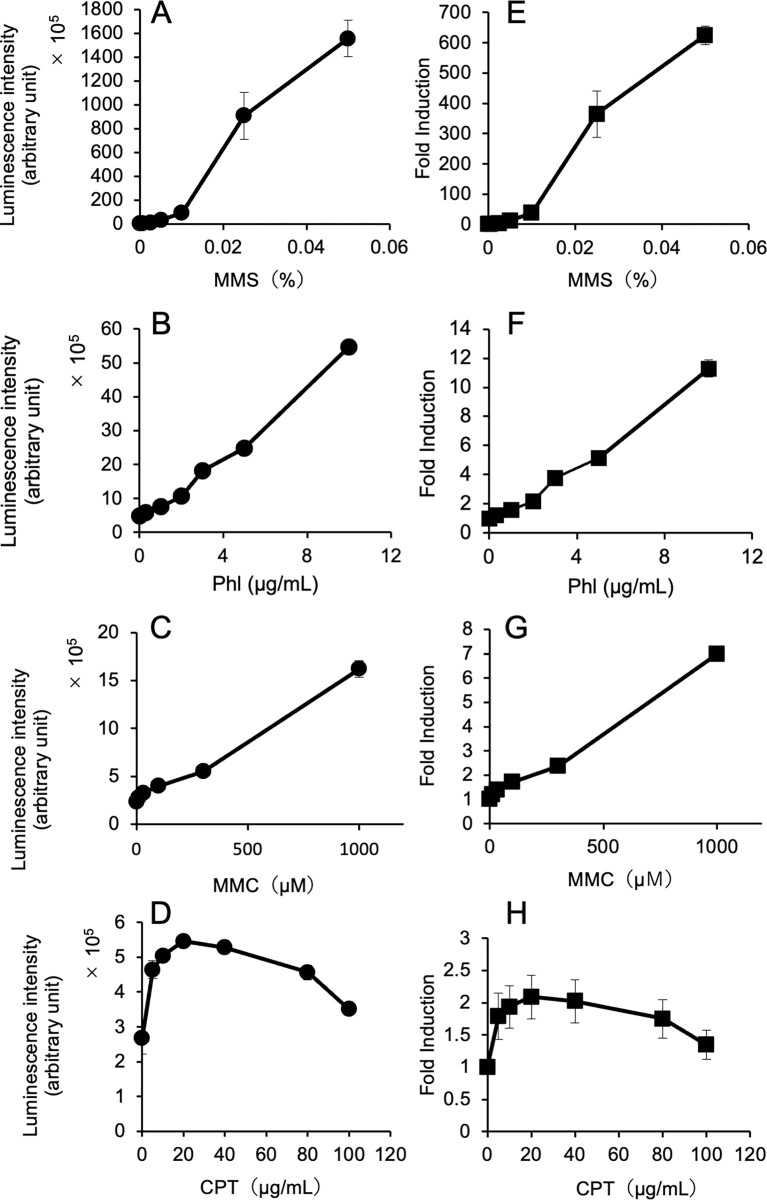
Nluc activity and fold induction in genotoxicant-treated yeast cells with a chromosomally integrated ^P^*RNR3-yNluc* gene. The luminescence intensity was normalized by the cell density and fold induction in the integrated reporter yeasts after exposure to the indicated concentrations of methyl methanesulfonate (MMS) (**A**, **E**), phleomycin (Phl) (**B**, **F**), mitomycin C (MMC) (**C**, **G**), and camptothecin (CPT) (**D**, **H**). The raw dataset for Fig 2 is shown in S5 Table.

Two types of reporter yeast strains carrying a multi-copy plasmid with a ^P^*RNR3*-linked *yNluc* gene and a chromosomally integrated reporter gene were generated, and the response of the *yNluc* gene in two yeast reporter strains in the presence of hydroxyurea (HU) was investigated. The luminescence intensity in yeasts with the reporter plasmid was more than 10-fold higher than that in the chromosomally integrated reporter yeasts (Fig 1A and 1B, S3 Table). However, significantly higher fold inductions were observed in the chromosomally integrated reporter strain treated with HU than those in the plasmid-based reporter strain (Fig 1C, S4 Table). Based on this observation, we investigated the responses of a chromosomally integrated ^P^*RNR3*-*yNluc* gene after exposure to four different types of genotoxic chemicals (MMS, Phl, MMC, and CPT). The luciferase activities (Fig 2A–2C, S5 Table) and fold inductions (Fig 2E–2G) were increased after treatment with MMS, Ph1, and MMC in a dose-dependent manner. In particular, yeast cells treated with MMS exhibited 100-fold inductions (Fig 2E). Conversely, CPT treatment did not cause a strong induction in luciferase activity in the reporter yeast cells (Fig 2D).

### Detection of oxidative stress using yeast strains carrying a *TRX2* promoter-linked *yNlucCP* gene on a multi-copy plasmid and a chromosome

**Fig 3:**
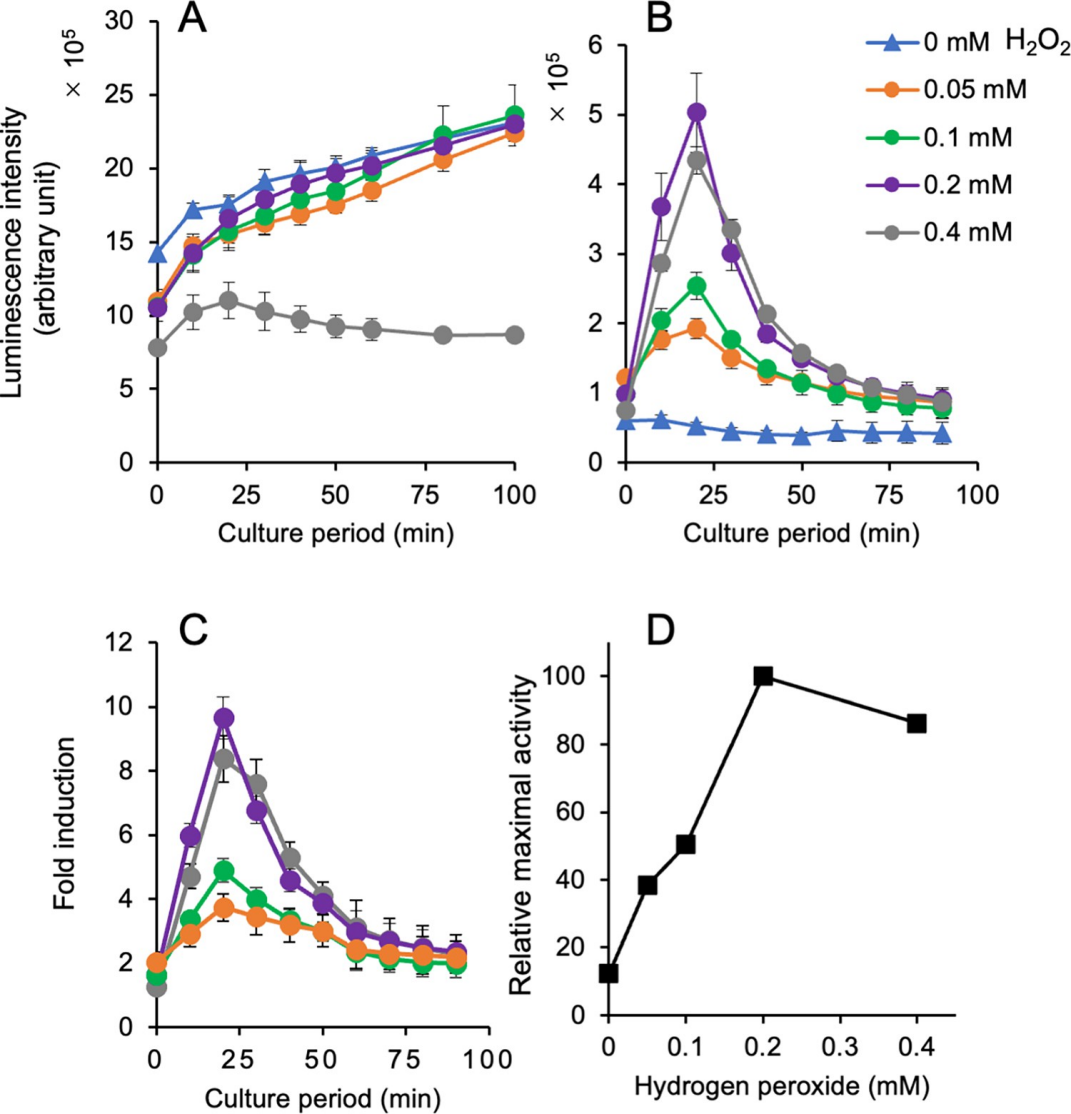
Response of ^P^*TRX2-yNlucCP* gene in yeast after exposure to hydrogen peroxide (H_2_O_2_). The luminescence intensities in yeast cells carrying a multi-copy reporter plasmid (**A**) and a chromosomally integrated reporter gene (**B**) were measured in the presence of H_2_O_2;_ the normalized intensity is shown with the standard deviations. (**C**) Fold inductions calculated from the activity of a panel (**B**) were plotted against the culture period. (**D**) The relative maximal luciferase activity was calculated as the percentage of each peak activity divided by the highest peak activity and plotted against the concentrations as shown in panel b with the standard deviations. The raw dataset for Fig 3 is shown in S6 Table.

We generated two yeast-based reporter assays using ^P^*TRX2-*linked unstable *yNluc* gene (*yNlucCP*) on a multi-copy plasmid and a chromosome V (*CAN1* locus) and investigated the response of the reporter gene in two strains treated with three oxidative chemicals: hydrogen peroxide (H_2_O_2_), *tert*-butyl hydroperoxide (*t*-BHP), and menadione. The maximal luminescence intensity in yeasts with a reporter plasmid was approximately 4.5-fold higher than the peak activity in a chromosomally integrated reporter yeast in the presence of 0.2 mM H_2_O_2_ (Fig 3A and 3B, S6 Table). Interestingly, the resultant induction profiles of the luciferase activity were different in the two reporter strains: the luciferase activity gradually increased in yeasts containing a plasmid with the same reporter gene (Fig 3A), whereas transient peaks of yNlucCP were detected in the chromosomally integrated reporter yeasts at 20 min after H_2_O_2_ addition (Fig 3B), as observed in our previous study [26]. The highest peak of fold induction was found in yeasts with a chromosomally integrated reporter gene in the presence of 0.2 mM H_2_O_2_ (Fig 3C). The “relative maximal activity” of luciferase was defined as the percentage of the highest peak activity at a tested concentration divided by the highest peak activity observed in the experiment in order to determine the induced levels of luciferase against the concentration of the oxidants. As shown in Fig 3D, luciferase induction occurred in a concentration-dependent manner up to 0.2 mM H_2_O_2_; the induction level decreased at a high concentration of 0.4 mM H_2_O_2_.

**Fig 4:**
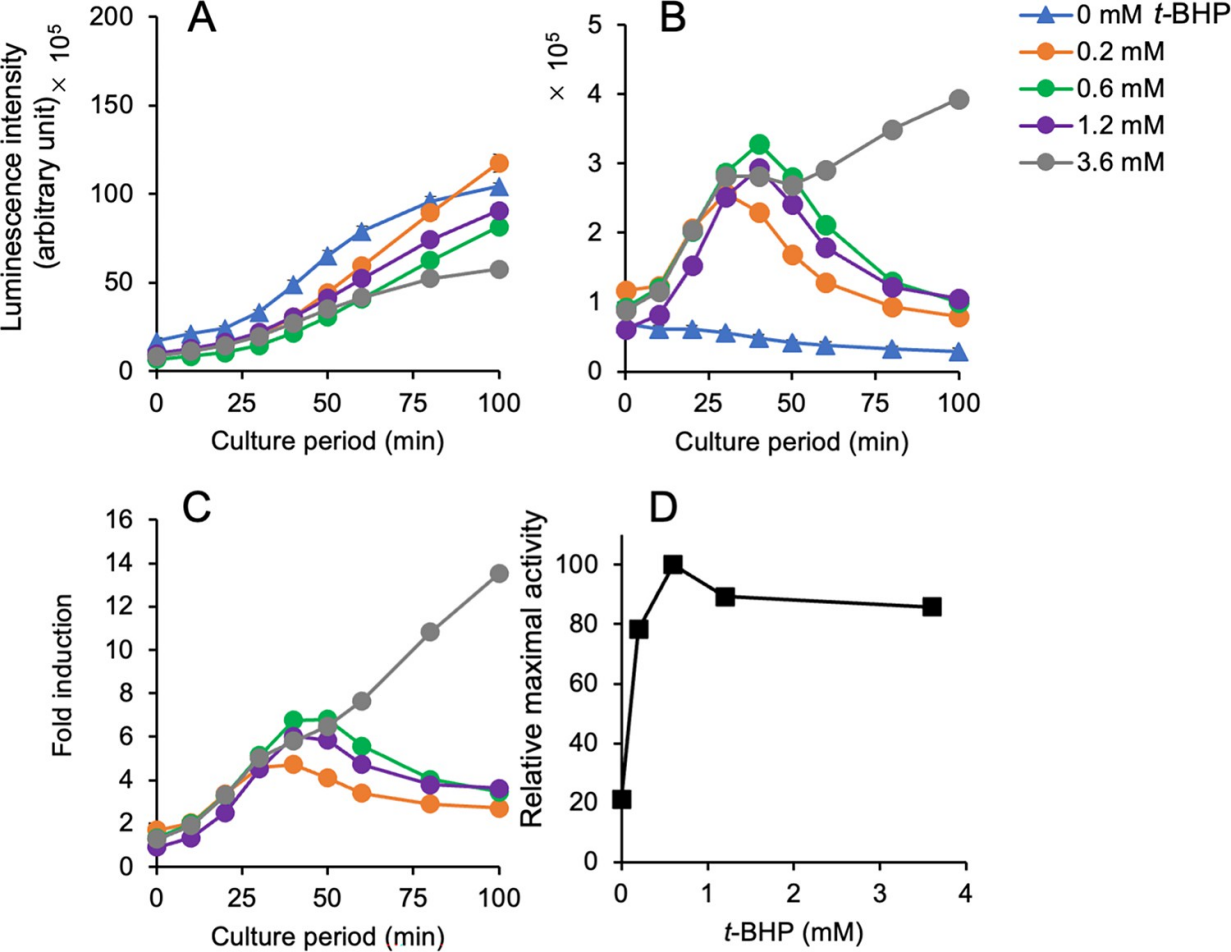
Response of a chromosomally integrated ^P^*TRX2-yNlucCP* gene in yeast after exposure to *tert*-butyl hydroperoxide (*t*-BHP). The luminescence intensities in yeast cells carrying a multi-copy reporter plasmid (**A**) and a chromosomally integrated reporter gene (**B**) were measured in the presence of the indicated concentrations of *t*-BHP. (**C**) Fold inductions calculated from the activity of a panel (**B**) are plotted against the culture period. (**D**) The relative maximal activities in yeasts treated with the indicated concentrations of *t*-BHP were calculated from the activity of a panel (**B**) and plotted against the concentrations with the standard deviations. The raw dataset for Fig 4 is shown in S7 Table.

**Fig 5:**
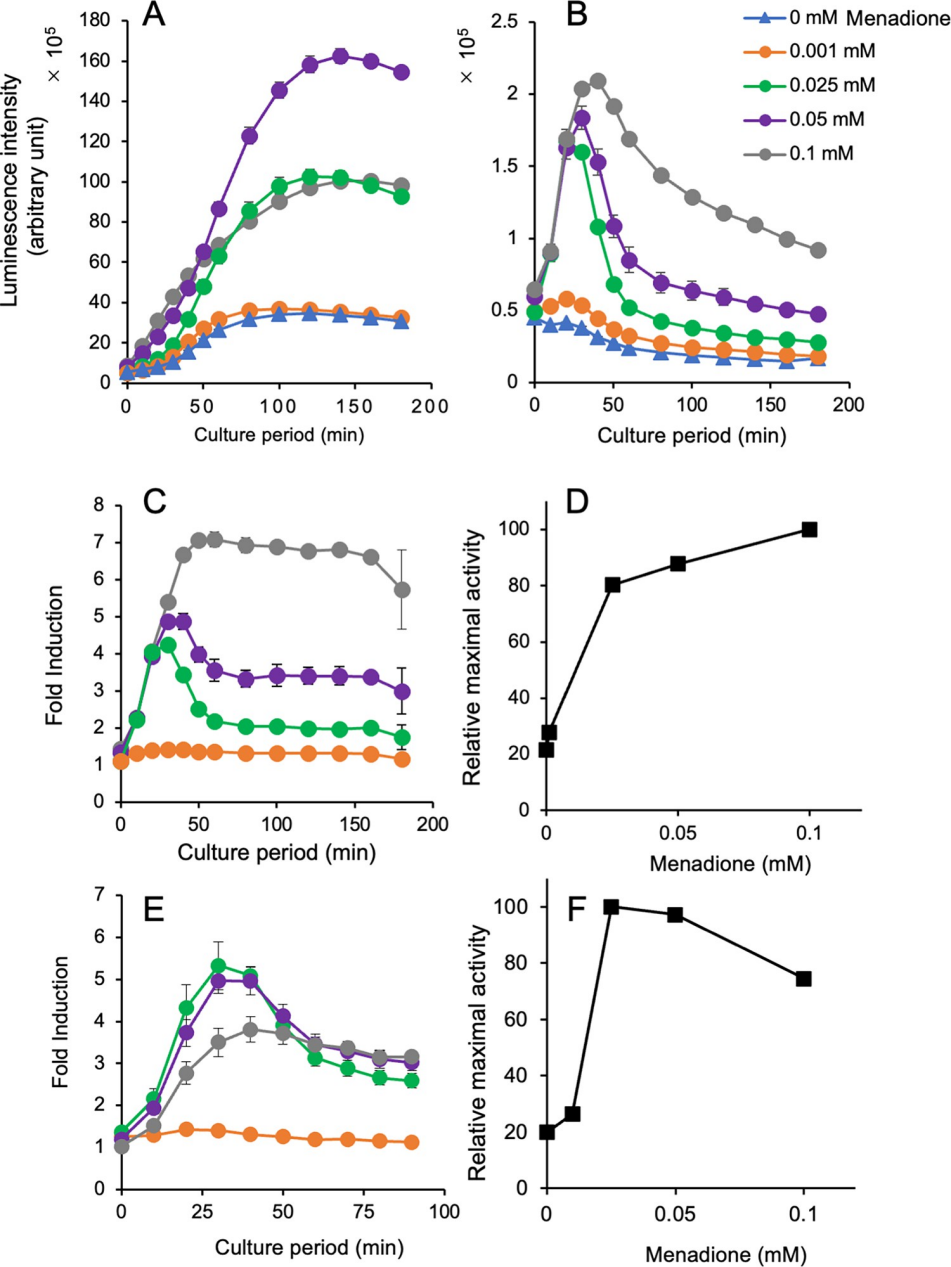
Response of a chromosomally integrated ^P^*TRX2-yNlucCP* gene in yeast exposed to menadione. The luminescence intensities in yeast cells carrying a multi-copy reporter plasmid (**A**) and a chromosomally integrated reporter gene (**B**) were measured in the presence of the indicated concentrations of menadione. (**C**) The fold inductions were calculated by the activity of a panel (**B**) during the culture period. (**D**) The relative maximal activities in yeasts treated with the indicated concentration of menadione were determined by the activity of a panel (**B**) and plotted against the concentrations with the standard deviations. (**E**) and (**F**) Fold induction and relative maximal activity plots, respectively, obtained from another experiment. The raw dataset for Fig 5 is shown in S8 Table.

Similar results were obtained from the assay using *t*-BHP (Fig 4, S7 Table). The luciferase peaks were not observed in the plasmid-based yeast reporter strain upto 100 min after treatment (Fig 4A). However, in the chromosomal integration reporter strain, transient peaks were found at 30–40 min after adding *t*-BHP (Fig 4B). Interestingly, the expression profile of luciferase at the highest concentration of *t*-BHP (3.6 mM) was different from those observed at other concentrations: Despite the presence of a peak around 30–40 min after treatment, the activity demonstrated a continuous increased in the presence of *t*-BHP even after 50 min after the culture period (Fig 4B). The relative maximal activity plateaued at 0.6 mM based on the induction peaks of the expression response caused by *t*-BHP (Fig 4D). Consistent observations were obtained from the assays using menadione-treated reporter yeasts (Fig 5, S8 Table). The expression of luciferase was gradually increased in yeasts with a reporter plasmid (Fig 5A), and transient peaks of luciferase expression were consistently observed in yeasts with an integrated reporter gene (Fig 5B). Luciferase activity measured until 200 min after menadione treatment indicating high levels of activity when the concentration of menadione was high (0.1 mM) in the chromosomally integrated reporter strain (Fig 5B). However, in the plasmid-based reporter assay, the levels of luciferase expression reached a plateau at 70–150 min; the plateau level increased when treated with 0.025 mM menadione and reached its maximum at 0.05 mM, followed by a decrease at 0.1 mM (Fig 5A). Menadione treatment clearly induced the expression of an integrated reporter gene as shown by the fold induction and relative maximal activity in this (Fig 5C and 5D) and another assay (Fig 5E and 5F).

### Response of a chromosomally integrated ^P^*TRX2-yNlucCP* gene after treatment with three oxidative chemicals

**Fig 6:**
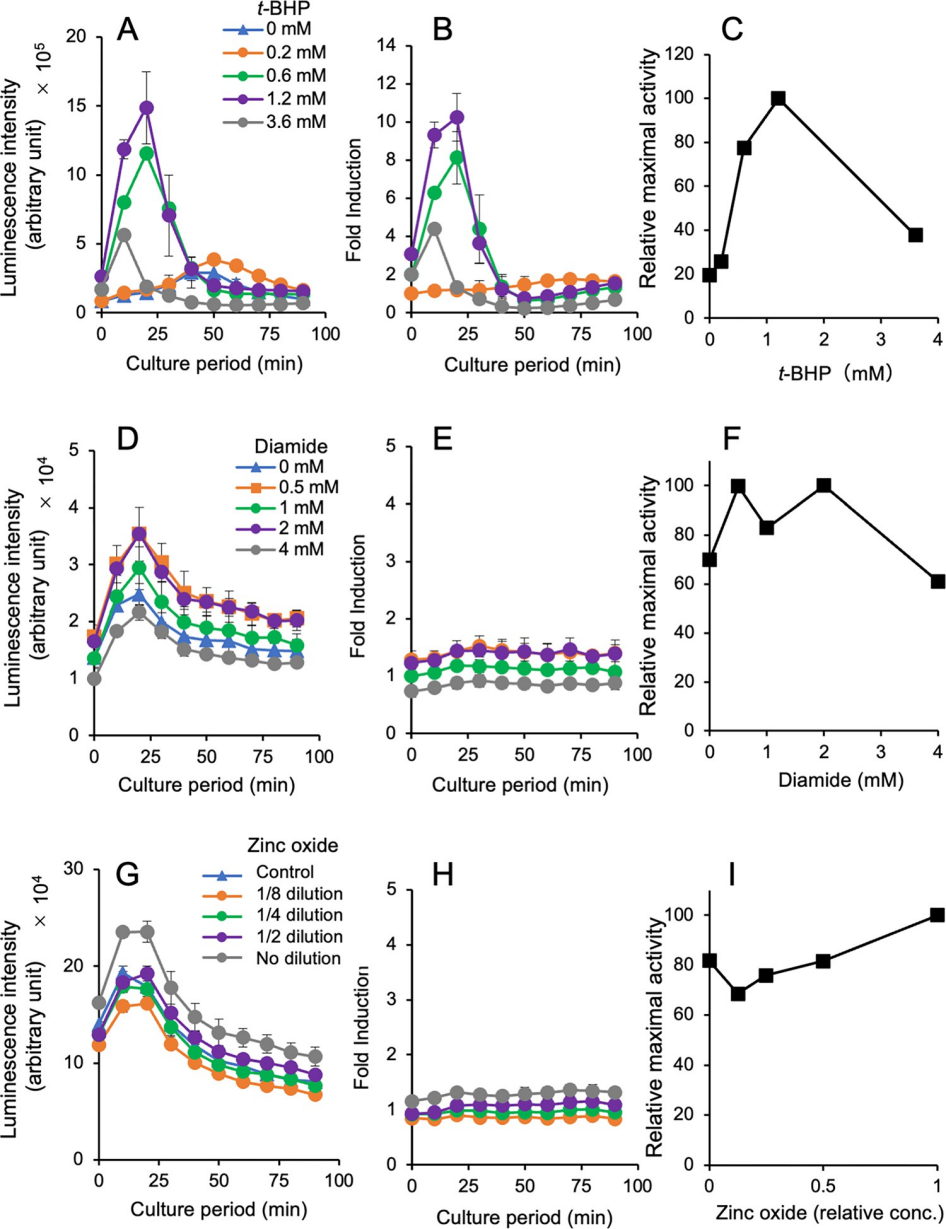
Response of a chromosomally integrated ^P^*TRX2-yNlucCP* gene in yeast cells treated with three chemicals. Normalized luciferase activities (**A, D, G**), fold inductions (**B, E, H**), and relative maximal activities (**C, F, I)** in yeasts after exposure to *t*-BHP (**A**–**C**), diamide (**D**–**F**), and zinc oxide suspension (**G**–**I**). The raw dataset for Fig 6 is shown in S9 Table.

Finally, we tried to detect the potential for oxidative stress caused by diamide and zinc oxide using yeast cells with an integrated ^P^*TRX2-yNlucCP* gene. Although diamide is known to cause oxidative stress, we failed to detect oxidative stress in our previous study [26]. Zinc oxide nanoparticles have been suggested to cause oxidative stress in animals [35]. In the present study, a typical transient expression of luciferase was found in reporter yeast cells exposed to *t*-BHP as a positive control in luciferase activity (Fig 6A, S9 Table) and fold induction (Fig 6B). The results of the relative maximal activity indicated luciferase induction with *t*-BHP (Fig 6C). However, the luciferase activities in yeast cells exposed to diamide (Fig 6D) and zinc oxide suspension (Fig 6G) were >20-fold lower than that in the *t*-BHP-treated cells. Although weak, luciferase peaks were found even in the absence of test chemicals. No increase in fold induction was observed, and the relative maximal activity did not show any chemical-mediated induction of unstable NanoLuc luciferase with diamide (Fig 6E and 6F) and zinc oxide suspension (Fig 6H and 6I).

## Discussion

### Detection of genotoxicity using yeast strains carrying a chromosomally integrated *RNR3* promoter-linked *yNluc* gene

In this study, we used a codon-optimized NanoLuc luciferase (yNluc) that produces strong luminescence as a reporter in yeast-based assays and a DNA damage-inducible promoter (^P^*RNR3*) to detect genotoxic chemicals. First, we investigated the response of a reporter gene with HU in two reporter systems: ^P^*RNR3*-linked *yNluc* gene on a multi-copy plasmid and integrated at the *CAN1* locus. As shown in Fig 1, high levels of luminescence intensity were observed in yeasts with a single copy of the reporter gene. Furthermore, a higher fold induction was observed in yeasts with an integrated reporter gene than in yeasts containing a reporter plasmid. While 10^3^–10^5^ counts normalized by *A*_600_ were observed as luciferase activity induced by MMS in the assay using yeasts with a chromosomally integrated ^P^*RNR3*-firefly luciferase (*luc2*) gene [26], 10^5^–10^8^ order of normalized counts was obtained in the MMS-treated yeast cells carrying an integrated ^P^*RNR3*-*yNluc* gene (Fig 2). Yeasts with an integrated reporter gene can be cultured and maintained in a non-selective medium; hence, they are suitable for genotoxicity assays compared to plasmid-based reporter assays. The assay using this reporter strain successfully detected four different types of DNA damage caused by chemicals: alkylated base damage (MMS), DNA strand breaks (Phl), DNA strand cross-links (MMC), and replication fork arrest (CPT) (Fig 2). The fold inductions observed in the assays with MMS and MMC are more than 10- and 2-fold higher than those in our previous study, which used a reporter plasmid with a ^P^*RNR3*-linked secretory luciferase gene [8]; nonetheless, the fold inductions in the system with Phl, HU, and CPT in the current study are almost comparable to the observations of our previous study [8]. Despite testing only five chemicals, our reporter assays cover the Ames-positive compounds (Table 3). Regarding the detectable concentrations of these chemicals, the lowest effective concentrations (LECs) in our yNluc reporter assay are comparable or slightly improved compared with the LECs in our previous assays [5,8,26], which used different reporters, as well as the yeast-based GreenScreen assay with a ^P^*RAD54*-linked *gfp* reporter gene [36].

**Table 3:** Detectable concentrations of genotoxic chemicals compared with our previous systems and other systems.

Promoter-reporter constructs in yeast or a bacterial assay for detecting genotoxicity	Exposure period for endpoint assay	Genotoxic chemicals tested					Reference
		Methylmethane sulfonate (MMS)	Hydroxyurea (HU)	Phleomycin (Phl)	Mitomycin C (MMC)	Camptothecin (CPT)	
^P^*RNR3-yNluc* gene integrated at the *CAN1* locus	8 h	0.0025%−0.05% (w/v)^a^ (0.0005%−0.05%)^b^	5−50 mM (2.5−50 mM)	0.3−10 μg/mL (0.3−10 μg/mL)	0.01−1 mM (0.01−1 mM); Low FI^c^	5−80 μg/mL (5−100 μg/mL); Low FI	Present study
^P^*RNR3- Cypridina luc* gene on a single copy plasmid	6, 24 h	0.0025%−0.1% (0.0025%−0.1%) (peak at 0.01% and decrease at >0.01%)	10−100 mM (3−100 mM)	0.3−10 μg/mL (0.3−10 μg/mL)	0.3−1 mM (0.03−1 mM) (at 24 h); Low FI	10−120 μg/mL (10−120 μg/mL) (at 6 h); low FI	Ochi et al. [8]
^P^*RNR3-lacZ* gene in a multicopy plasmid	6 h	0.0025%−0.1% (0.0025%−0.1%) (peak at 0.02%)	Not tested	Not tested	Not tested	Not detected at 5−160 μg/mL	Ichikawa and Eki [5]
^P^*RNR3-luc2* gene on a multicopy plasmid	6, 24 h	0.0025%−0.02% (0.001%−0.02%)	Not tested	Not tested	Not tested	Not tested	Suzuki et al. [26]
^P^*RAD54-gfp* gene in a multicopy plasmid (GreenScreen assay)	16−20 h (overnight)	1.02 μg/mL (0.0001%) (LEC)^d^	593 μg/mL (7.8 mM) (LEC)	12.5 μg/mL (LEC)	200 μg/mL (0.6 mM) (LEC)	Not tested	Cahill et al. [36]
Ames test	−	Yes^e^	Yes	Not found	Yes	Not found	Madia et al. [37]

^a^Range of detectable concentrations

^b^range of tested concentrations

^c^low FI: Low fold induction

^d^LEC: Lowest effective concentrations detected in the GreenScreen assay

^e^Yes: Ames positive.

These findings suggest that yeast strains carrying an integrated ^P^*RNR3*-*yNluc* gene can be used as an alternative to the currently used yeast-based genotoxicity assays, which employ a reporter plasmid. In the future, using DNA repair-deficient mutants as hosts can make this genotoxicity assay more sensitive against low concentrations of genotoxicants, as shown in several studies [9,12,38], including ours [8,26].

### Detection of oxidative chemicals by yeast strains carrying a *TRX2* promoter-linked *yNlucCP* gene

In our previous study, we developed a yeast-based assay using a multi-copy plasmid carrying a ^P^*TRX2-*driven unstable firefly luciferase (*luc2CP*) gene and detected oxidative stress via the transient expression of luciferase activity 60–90 min after treatment with the oxidants [26]. However, in the current study, the results of the assay using a multi-copy plasmid with the ^P^*TRX2-yNlucCP* gene were different from those reported previously [26]. Nluc activities after treatment with oxidants gradually increased during the culture period without transient peaks of activity (Figs 3A, 4A and 5A). The reason for these discrepant observations between assays using a plasmid-based reporter with destabilized firefly and NanoLuc luciferases remains unknown.

Yeast cells carrying a chromosomally integrated ^P^*TRX2-yNlucCP* gene effectively sensed three representative oxidants (H_2_O_2_, *t*-BHP, and menadione) but not diamide and zinc oxide (Figs 3–6). These results from four oxidants, except for zinc oxide, are consistent with our previous observations in the assay with a Luc2CP reporter [26]. Additionally, the maximal levels of luciferase induction in each assay (5–15 fold induction) are comparable to those in our previous study (3–10) [26]. High levels of luminescence intensity derived from unstable NanoLuc luciferase (NlucCP) contribute to the transient expression in assays using yeasts with a single copy of the chromosomally integrated reporter gene. We summarized the detectable concentrations in this reporter system compared with our previous system, which used yeast strains with a Luc2CP reporter on a multicopy plasmid, and the yeast-based assay using a catalase 1 (*CTT1*) promoter–driven unstable luciferase gene developed by other researchers [23] (Table 4). In essence, the detectable oxidants and the range of oxidant concentrations are comparable in our real-time luciferase assays with reporter yeasts, Specifically, hydrogen peroxide, *t*-BHP, and menadione can be detected, but diamide cannot. Additionally, the reporter assay using yeasts with an oxidative stress–responsive *CTT1* promoter–linked luciferase gene was able to detect >100 μM hydrogen peroxide, as well as >30 μM menadione [23]. Furthermore, the recombinant yeast strain BioS-OS1/2, featuring an oxidative stress–inducible GFP reporter, detected 0.3 mM H_2_O_2_ [24]. The question arises as to what concentrations of oxidants should be detectable in the assay. Previous studies revealed the 50% effective concentrations (EC_50_) of hydrogen peroxide that caused toxicity (viability, cell death, or apoptosis) in mammalian cells: EC_50_ values of 100 and 500 μM in mouse endothelial cell lines [39], <100 μM in rat neuronal B50 cells [40], 1 mM in human neuroblastoma SH-SY5Y cells [41], and 30–500 μM (depending on the exposure duration) in rat C6 glioma cells [42]. It has been reported that adverse effects in neurons were induced by 12−500 μM hydrogen peroxide [43], and 25−50 μM hydrogen peroxide led to apoptosis in human Jurkat T-lymphocytes [44]. Our reporter assays can detect 30−50 μM hydrogen peroxide at the lowest concentrations covering the EC_50_ values mentioned above. However, these assays are currently unable to effectively detect lower concentrations of hydrogen peroxide at levels <10 μM. In the future, further improvements in the reporter assays may be necessary, including using yeast strains with disrupted genes involved in cell permeability to enhance sensitivity to oxidants.

**Table 4:** Detectable concentrations of oxidants in three reporter systems.

Oxidant tested	^P^*TRX2-yNlucCP* gene integrated at the *CAN1* locus	^P^*TRX2-luc2CP* gene in a multicopy plasmid [26]	^P^*CTT1-lucCP* gene in a single-copy plasmid [23]
H_2_O_2_	0.05−0.4 mM^a^ (0.05−0.4 mM)^b^	0.03−0.1 mM (0.01−0.4 mM)	0.1−0.8 mM (0.025−2 mM)
*t*-BHP	0.2−3.6 mM (0.2−3.6 mM)	0.03−3.6 mM (0.003−3.6 mM)	Not tested
Menadione	0.025−0.1 mM (0.001−0.1 mM)	0.025−0.1 mM (0.01−1 mM)	0.03−0.17 mM (0.02−0.17 mM)
Diamide	Not detected (0.5−4 mM)	Not detected (0.01−5 mM)	Not tested
Diethyl maleate	Not tested	Not detected (0.1−10 mM)	Not tested
Saturated ZnO suspension	Not detected (1/8−no dilution)	Not tested	Not tested

^a^Range of detectable concentrations

^b^range of tested concentrations.

In this study, we observed a new finding in the assay for oxidative stress. High levels of luciferase activities increased or tailed after the peak in the assays with *t*-BHP and menadione (Figs 4B and 5B). The oxidant-triggered transcriptional induction of yeast genes, including *TRX2*, which encodes most antioxidants and components of the cellular thiol-reducing pathway, is regulated by a leucine zipper transcription factor Yap1p [27,45,46]. Under oxidative stress-free conditions, Yap1p localizes in the cytoplasm due to rapid transport by the nuclear export receptor Crm1p. Oxidative stress causes redox-dependent conformational changes via intramolecular bond formations of the cysteine residues at the C- and N-terminus regions in Yap1p, thereby avoiding interactions between Yap1p and Crm1p. The resultant nuclear accumulation of Yap1p activates the transcription of the oxidative stress-responsive genes [47]. The transient activation of the *TRX2* promoter by oxidants may be caused by the rapid recovery of the redox state of Yap1p via the overlapped antioxidants and the redox regulation systems. Based on this idea, the phenomena described above were likely caused by a delayed recovery of the redox state of Yap1p due to the excess amounts of oxidants; these phenomena were commonly observed in the presence of high concentrations of the oxidants (3.6 mM *t*-BHP in Fig 4B, and 0.1 mM menadione in Fig 5B).

Oxidative stress with diamide and zinc oxide was poorly detected in the current study (Fig 6). H_2_O_2_, *t*-BHP, and menadione are producers of ROS *in vivo*, whereas diamide causes oxidative stress via the oxidation of the sulfhydryl groups in glutathione. The different levels of luciferase induction observed in the two groups of oxidants could be their distinct mechanisms of action. Zinc oxide nanoparticles are widely used in industrial applications, including cosmetics; however, recent studies show that zinc oxide nanoparticles induce oxidative stress via ROS production in zebrafish [48] and human hepatic cells [49]. Although oxidative stress was not detected in our yeast-based reporter assay using the zinc oxide suspension, further tests are required to confirm the findings using different types of zinc oxide nanoparticles. The reporter system used in the current study was based on transient expression caused by oxidative stress, which is measured by the real-time reporter assay using yeasts with a stress-responsible promoter-linked destabilized reporter gene. The transient expression of the yeast genes is caused by environmental or chemical stress [23,50]. Therefore, similar yeast-based reporter systems have been developed; for example, for the selective detection of drugs [51]. Using an unstable NanoLuc luciferase as a reporter could improve these yeast-based real-time assays by virtue of the high levels of luminescence intensity.

## Conclusion

In this study, yeast-based assays were developed for detecting chemically-induced genotoxicity and oxidative stress using NanoLuc luciferase as a reporter. Despite the use of a single copy of the reporter gene, the assays using yeasts with chromosomally integrated *RNR3*- and *TRX2* promoter-driven stable and unstable *Nluc* gene successfully assessed the genotoxicity and oxidative stress, respectively, more effectively than the reporter plasmid-based assays. Integrated reporter strains are easily maintained in a non-selective medium; therefore, reporter assays with these strains can be used as a convenient tool for screening potential genotoxic or oxidative chemicals.

## Supporting information

S1 Fig Nucleotide sequence of the codon-optimized *yNlucCP* gene. Nucleotide sequence of the codon-optimized yNlucCP gene was shown.
(PDF)

S1 Table PCR primers and nucleotide sequences used in this study. (PDF)

S2 Table Primer sets used for confirmation of chromosomal integration by colony PCR. (PDF)

S3 Table Raw dataset for Fig 1. (PDF)

S4 Table Statistical analysis by Student’s *t*-test for two reporter systems. (PDF)

S5 Table Raw dataset for Fig 2. (PDF)

S6 Table Raw dataset for Fig 3. (PDF)

S7 Table Raw dataset for Fig 4. (PDF)

S8 Table Raw dataset for Fig 5. (PDF)

S9 Table Raw dataset for Fig 6. (PDF)
