# Supplementary material for: Sensing chemical-induced genotoxicity and oxidative stress via yeast-based reporter assays using NanoLuc luciferase
Source: PLoS One. 2023 Nov 22;18(11):e0294571. doi: 10.1371/journal.pone.0294571 (PMC10664910; doi:10.1371/journal.pone.0294571)
Supplement: S1 Fig — Nucleotide sequence of the codon-optimized yNlucCP gene was shown. (PDF) [file pone.0294571.s001.pdf]

ATGGTTTTTACTTTAGAAGATTTTGTTGGTGATTGGCGTCAAACTGCTGGTTATAATTTAGATCAAG

TTTTAGAACAAGGTGGTGTTTCTTCTTTATTTCAAAATTTAGGTGTTTCTGTTACTCCTATTCAACG

TATTGTTTTATCTGGTGAAAATGGTTTAAAAATTGATATTCATGTTATTATTCCTTATGAAGGTTTA

TCTGGTGATCAAATGGGTCAAATTGAAAAAATTTTTAAAGTTGTTTATCCTGTTGATGATCATCATT

TTAAAGTTATTTTACATTATGGTACTTTAGTTATTGATGGTGTTACTCCTAATATGATTGATTATTT

TGGTCGTCCTTATGAAGGTATTGCTGTTTTTGATGGTAAAAAAATTACTGTTACTGGTACTTTATGG

AATGGTAATAAAATTATTGATGAACGTTTAATTAATCCTGATGGTTCTTTATTATTTCGTGTTACTA

TTAATGGTGTTACTGGTTGGCGTTTATGTGAACGTATTTTAGCTAATTCTCATGGTTTTCCTCCTGA

AGTTGAAGAACAAGCTGCTGGTACTTTACCTATGTCTTGTGCTCAAGAATCTGGTATGGATCGTCAT

CCTGCTGCTTGTGCTTCTGCTCGTATTAATGTTTAAATGATTACGCCAAGCTTGAAGGATCATCTCA

TCCACAATGTCCACAAAGAGGAGCACGCTCATGCCCACAACAAGATCGATGACGATGACAAA
