# Supplementary material for: Sensing chemical-induced genotoxicity and oxidative stress via yeast-based reporter assays using NanoLuc luciferase
Source: PLoS One. 2023 Nov 22;18(11):e0294571. doi: 10.1371/journal.pone.0294571 (PMC10664910; doi:10.1371/journal.pone.0294571)
Supplement: S1 Table — (PDF) [file pone.0294571.s002.pdf]

S1 Table. PCR primers and nucleotide sequences used in this study.

Primer	Nucleotide sequence (5' to 3')	Use
pESChis2DSma-RNR3P-IFF	CTCACTATAGGGCCCTTGTCGTCGCTGGAGGCGTACTAG	Preparation of pESC-HISΔ*GAL1/10*-^{P}*RNR3-yNluc* plasmid
RNR3P-yNluc25-R	CAAAATCTTCTAAAGTAAAAACCATTTGTGTGGGAGTATTTGATTTATTGC	
yNluc-5'-F	ATGGTTTTTACTTTAGAAGATTTTG	
pESChis2DSma-yNlucend-IFR	TCCATGTCGACGCCCTTAAGCTAAAATACGTTCACATAAACG	
pESChis2DSma-TRX2P500-IFF	CTCACTATAGGGCCCAACATCCAGACTTTTACGGGTGG	Preparation of pESC-HISΔ*GAL1/10*-^{P}*TRX2-yNlucCP* plasmid
TRX2P500-yNluc25-R	ATATCTTTAAATAACACATCAATAATGGTTTTTACTTTAGAAGATTTTG	
yNluc-5'-F	ATGGTTTTTACTTTAGAAGATTTTG	
pESChis2DSma-yNlucPEST-IFR	TCCATGTCGACGCCCTTAAACATTAATACGAGCAGAAGC	
5'-CAN1-F	AATAGGGCGAACTTGAAGAATAACC	Preparation of yeast strains with chromosomally integrated reporter genes
5-CAN1-R_5F2_30tail	AGTTGCGCAGCCTGAATGGCGAATGGACGCAAATTTTCATTGATAGAGACAACTG	
3-CAN1-F_3R3_30tail	CGCTCGGTCGGCTGCGGCGAGCGGTAATATGACGTTTTATTACCTTTGATC	
3'-CAN1-R	TACTTGAAGGTCTGAAGGAGTTTCA	
TADH1-5F2-25CAN1-Rtail	CAGTTGTCTCTATCAATGAAAATTTGCGTCCATTCGCCATTCAGGCTGCG	
TCYC1-3R3-25CAN1-Ftail	GATCAAAGGTAATAAAACGTCATATTACCGCTCGCCGCAGCCGAACGACC	
CAN1orf-F	ATTTAAAGCTAAATTAATGCCCGG	Confirmation of chromosomal integration for ^{P}*RNR3-yNluc* DNA and ^{P}*TRX2-yNlucCP* DNA
CAN1_dg rv	GGTTCTAGGTTCGGGTGACG	
yNluc-SQR1	CATTTGATCACCAGATAAACC	
yNluc-5'-F	ATGGTTTTTACTTTAGAAGATTTTG	
RNR3-SQ516over-F	GAGCAAGCCCTCGTTCTTGG	
TRX2P-SQF1	GTGAGAGTCAGTGAAGAAAGTC	
