# Supplementary material for: Sensing chemical-induced genotoxicity and oxidative stress via yeast-based reporter assays using NanoLuc luciferase
Source: PLoS One. 2023 Nov 22;18(11):e0294571. doi: 10.1371/journal.pone.0294571 (PMC10664910; doi:10.1371/journal.pone.0294571)
Supplement: S2 Table — (PDF) [file pone.0294571.s003.pdf]

**S2 Table. Primer sets used for confirmation of chromosomal integration by colony PCR.**

| Name of primer set | Primer set for colony PCR | Wild-type *CAN1* allele (bp) | $^{P}$*RNR3-yNluc*-integrated allele (bp) |
|---|---|---|---|
| Primer set A | CAN1orf-F/CAN1_dg rv | 545 | NA |
| Primer set B | yNluc-5'-F/CAN1_dg rv | NA | 1243 |
| Primer set C | RNR3-SQ516over-F/yNluc-SQR1 | NA | 664 |
| | | Wild-type *CAN1* allele (bp) | $^{P}$*TRX2-yNlucCP*-integrated allele (bp) |
| Primer set A | CAN1orf-F/CAN1_dg rv | 545 | NA |
| Primer set B | RAD10_dg fw/RAD10_dg rv | NA | 1351 |
| Primer set D | TRX2P-SQF1/yNluc-SQR1 | NA | 571 |

Primer sets used for confirmation of integrated alleles by colony PCR are shown with the expected sizes of the colony PCR products from *CAN1* and integrated alleles. Primer sets C and D were used to confirm the presence of the reporter genes. NA: No amplification
