# Supplementary material for: Sensing chemical-induced genotoxicity and oxidative stress via yeast-based reporter assays using NanoLuc luciferase
Source: PLoS One. 2023 Nov 22;18(11):e0294571. doi: 10.1371/journal.pone.0294571 (PMC10664910; doi:10.1371/journal.pone.0294571)
Supplement: S3 Table — (PDF) [file pone.0294571.s004.pdf]

S3 Table. Raw dataset for Fig 1.

Reporter system	Hydroxyurea conc. (mM)	0	2.5	5	10	20	50
Plasmid-based ^{P}*RNR3-yNluc* reporter assay	Luminescence intensity (Mean)	42863200	42426406	49056211	65299524	66275825	126796230
	Luminescence intensity (SD)	1184818	1927033	1546250	1090302	13480128	7315648
	Fold induction (Mean)	(1.0)	0.99	1.14	1.52	1.55	2.96
	Fold induction (SD)		0.06	0.03	0.05	0.32	0.22
Chromosomally integrated ^{P}*RNR3-yNluc* reporter assay	Luminescence intensity (Mean)	802928	832725	1452659	3199205	5218393	8921725
	Luminescence intensity (SD)	18021	38365	30340	134610	323382	1080672
	Fold induction (Mean)	(1.0)	1.04	1.81	3.99	6.51	11.15
	Fold induction (SD)		0.07	0.07	0.25	0.52	1.59

Yeast strains with two reporter constructs for genotoxicity were cultured with the indicated concentrations of hydroxyurea for 8 h to measure the absorbance at 600 nm (A_{600}) and luminescence intensity. The raw data, including the mean and standard deviation (SD) of luminescence intensity corrected by the A_{600} value and fold inductions, are shown for each assay (n = 3).
