# Supplementary material for: Sensing chemical-induced genotoxicity and oxidative stress via yeast-based reporter assays using NanoLuc luciferase
Source: PLoS One. 2023 Nov 22;18(11):e0294571. doi: 10.1371/journal.pone.0294571 (PMC10664910; doi:10.1371/journal.pone.0294571)
Supplement: S4 Table — (PDF) [file pone.0294571.s005.pdf]

S4 Table. Statistical analysis by Student's *t*-test for two reporter systems.

HU concentration	p-value
2.5 mM	0.01040
5 mM	**0.00420**
10 mM	**0.00412**
20 mM	**0.00498**
50 mM	0.01553

Statistical significance of fold induction was tested by a two-tailed paired Student's t-test between the multi-copy plasmid-based and chromosome integrated reporter strains treated with hydroxyurea (HU). P-values in bold <0.01.
