# Supplementary material for: Sensing chemical-induced genotoxicity and oxidative stress via yeast-based reporter assays using NanoLuc luciferase
Source: PLoS One. 2023 Nov 22;18(11):e0294571. doi: 10.1371/journal.pone.0294571 (PMC10664910; doi:10.1371/journal.pone.0294571)
Supplement: S5 Table — (PDF) [file pone.0294571.s006.pdf]

S5 Table. Raw dataset for Fig 2.

Methyl methanesulfonate (MMS)

MMS conc. (%[w/v])	0	0.0005	0.0025	0.005	0.01	0.025	0.05
Luminescence intensity (Mean)	248654	274141	948085	3192578	9220854	90722756	155638434
Luminescence intensity (SD)	11845	12302	97727	107803	353475	19754161	15172978
Fold induction (Mean)	(1.0)	1.11	3.82	12.86	37.11	364.30	624.48
Fold induction (SD)		0.09	0.39	0.60	0.87	77.14	30.26

Phleomycin (Phl)

Phl conc. (μg/mL)	0	0.3	1	2	3	5	10
Luminescence intensity (Mean)	484852	586504	766879	1060927	1810893	2485244	5466267
Luminescence intensity (SD)	24892	42646	19767	109353	36364	136032	207513
Fold induction (Mean)	(1.0)	1.21	1.59	2.19	3.75	5.14	11.30
Fold induction (SD)		0.03	0.08	0.16	0.26	0.43	0.58

Mitomycin C (MMC)

MMC conc. (μM)	0	10	30	100	300	1000
Luminescence intensity (Mean)	232320	277545	324153	396941	549856	1622495
Luminescence intensity (SD)	12668	19740	63205	20789	46653	86903
Fold induction (Mean)	(1.0)	1.19	1.39	1.71	2.37	6.98
Fold induction (SD)		0.04	0.20	0.06	0.16	0.01

Camptothecin (CPT)

CPT conc. (μg/mL)	0	5	10	20	40	80	100
Luminescence intensity (Mean)	268480	463845	504081	545432	527854	456232	351119
Luminescence intensity (SD)	46738	25950	9040	4995	8550	20565	5344
Fold induction (Mean)	(1.0)	1.79	1.93	2.09	2.02	1.75	1.35
Fold induction (SD)		0.36	0.33	0.34	0.33	0.30	0.22

Yeast strains with a chromosomally integrated ^{P}*RNR3-yNluc* reporter gene for genotoxicity were cultured with the indicated concentrations of each chemical for 8 h to measure the absorbance at 600 nm (A_{600}) and luminescence intensity. The raw data, including the mean and standard deviation (SD) of luminescence intensity corrected by the A_{600} value and fold induction, are presented in each assay (n = 3).
