# Supplementary material for: Sensing chemical-induced genotoxicity and oxidative stress via yeast-based reporter assays using NanoLuc luciferase
Source: PLoS One. 2023 Nov 22;18(11):e0294571. doi: 10.1371/journal.pone.0294571 (PMC10664910; doi:10.1371/journal.pone.0294571)
Supplement: S6 Table — (PDF) [file pone.0294571.s007.pdf]

S6 Table. Raw dataset for Fig 3.

	Culture period (min)	0	10	20	30	40	50	60	80	100
	Luminescence intensity (Mean)									
	0 mM hydrogen peroxide	1426162	1718529	1755474	1909642	1963776	2009949	2084857	2203158	2311139
	0.05 mM hydrogen peroxide	1094696	1475486	1552327	1624846	1687191	1752049	1849242	2058544	2239618
	0.1 mM hydrogen peroxide	1057079	1408488	1573619	1674037	1789405	1843932	1972706	2223490	2360697
Plasmid-based ^{P}*TRX2-yNlucCP* reporter assay	0.2 mM hydrogen peroxide	1051464	1423876	1655113	1787675	1890337	1964881	2018608	2152891	2298609
	0.4 mM hydrogen peroxide	778180	1022705	1101133	1026794	974175	925790	904488	866969	868051
	Luminescence intensity (SD)									
	0 mM hydrogen peroxide	42087	47004	53047	79539	91101	55870	52410	62391	35169
	0.05 mM hydrogen peroxide	80516	57800	93853	72109	70517	60021	71437	80559	56432
	0.1 mM hydrogen peroxide	97716	101422	129371	128078	110247	135513	132705	200789	208579
	0.2 mM hydrogen peroxide	89990	130349	163854	133784	153500	119061	102443	91015	87634
	0.4 mM hydrogen peroxide	39340	118745	125720	128502	89152	76083	73047	35443	31988

	Culture period (min)	0	10	20	30	40	50	60	70	80	90
	Luminescence intensity (Mean)										
	0 mM hydrogen peroxide	60482	61365	52173	44663	40694	38834	45489	43457	43422	41945
	0.05 mM hydrogen peroxide	121730	176362	192895	151265	127574	115220	103560	94720	91824	86362
	0.1 mM hydrogen peroxide	98672	204905	253963	176754	134550	114651	98567	86999	81222	77538
	0.2 mM hydrogen peroxide	98470	367735	503173	300772	184417	148874	124435	108405	98562	91042
	0.4 mM hydrogen peroxide	74922	286442	433855	335246	213069	157381	128450	107443	97056	86865
	Luminescence intensity (SD)										
	0 mM hydrogen peroxide	7239	6445	5449	5623	5345	5215	15264	14957	15616	15434
	0.05 mM hydrogen peroxide	12212	13541	14280	16684	15705	17753	20489	22017	23279	21785
	0.1 mM hydrogen peroxide	8051	16409	19198	7338	7308	8647	8417	10870	13466	14697
	0.2 mM hydrogen peroxide	9993	48509	57161	24336	11460	9565	11250	12465	12831	13747
Chromosomally integrated ^{P}*TRX2-yNlucCP* reporter assay	0.4 mM hydrogen peroxide	3085	11055	19540	13591	11424	7831	5193	6025	6123	7152
	Fold induction (Mean)										
	0.05 mM hydrogen peroxide	2.04	2.91	3.73	3.45	3.19	3.01	2.42	2.29	2.25	2.18
	0.1 mM hydrogen peroxide	1.64	3.36	4.90	4.00	3.35	2.99	2.35	2.15	2.01	1.98
	0.2 mM hydrogen peroxide	1.63	5.99	9.66	6.78	4.58	3.88	2.96	2.68	2.47	2.35
	0.4 mM hydrogen peroxide	1.25	4.71	8.39	7.60	5.30	4.11	3.11	2.70	2.47	2.28
	Fold induction (SD)										
	0.05 mM hydrogen peroxide	0.30	0.39	0.43	0.57	0.52	0.49	0.59	0.47	0.58	0.51
	0.1 mM hydrogen peroxide	0.06	0.24	0.38	0.37	0.32	0.29	0.57	0.43	0.44	0.43
	0.2 mM hydrogen peroxide	0.06	0.37	0.66	0.42	0.35	0.33	0.67	0.55	0.58	0.56
	0.4 mM hydrogen peroxide	0.14	0.39	0.72	0.77	0.48	0.43	0.85	0.68	0.69	0.59

Hydrogen peroxide conc. (mM)	0	0.05	0.1	0.2	0.4
Relative maximal activity	12.20	38.34	50.47	100.00	86.22

Yeast strains containing two reporter constructs for sensing oxidative stress were cultured with the indicated concentrations of hydrogen peroxide. Luminescence intensity in each sample was measured at the indicated time intervals. The raw data, including the mean and standard deviation (SD) of luminescence intensity corrected by A600 value (measured at time 0), for two reporter assays with or without hydrogen peroxide are shown for the indicated culture periods (n = 3). Additionally, the mean and SD of fold induction and the relative maximal activity (refer to the Materials and Methods section) in the chromosomally integrated reporter system are shown.
