# Supplementary material for: Sensing chemical-induced genotoxicity and oxidative stress via yeast-based reporter assays using NanoLuc luciferase
Source: PLoS One. 2023 Nov 22;18(11):e0294571. doi: 10.1371/journal.pone.0294571 (PMC10664910; doi:10.1371/journal.pone.0294571)
Supplement: S7 Table — (PDF) [file pone.0294571.s008.pdf]

S7 Table. Raw dataset for Fig 4.

	Culture period (min)	0	10	20	30	40	50	60	80	100
	Luminescence intensity (Mean)									
	0 mM *t*-BHP	1702469	2110820	2424848	3325045	4872970	6519754	7904102	9596538	10438844
	0.2 mM *t*-BHP	912326	1170288	1455918	2035540	3086478	4437805	5942583	8951952	11765101
	0.6 mM *t*-BHP	659038	838479	1057759	1459815	2153867	3067542	4089975	6237594	8163650
Plasmid-based ^{P}*TRX2-yNlucCP* reporter assay	1.2 mM *t*-BHP	990791	1289474	1643321	2195711	3077777	4131031	5230953	7427960	9068085
	3.6 mM *t*-BHP	850104	1111629	1453707	1972476	2713357	3521591	4180488	5236960	5765985
	Luminescence intensity (SD)									
	0 mM *t*-BHP	184928	162234	141637	140937	254298	279379	283014	261955	208625
	0.2 mM *t*-BHP	55379	56779	47209	96047	198980	255365	307400	421284	507463
	0.6 mM *t*-BHP	42918	44846	52927	74008	151954	214167	285438	310755	395784
	1.2 mM *t*-BHP	13107	14680	39869	70963	114454	217816	298932	341138	368171
	3.6 mM *t*-BHP	32404	48102	15003	41591	65491	115953	110362	202352	207494

	Culture period (min)	0	10	20	30	40	50	60	80	100
	Luminescence intensity (Mean)									
	0 mM *t*-BHP	68897	61213	61555	55940	48689	41371	38016	32263	29179
	0.2 mM *t*-BHP	116762	123662	206804	257034	229766	169252	128524	93205	78898
	0.6 mM *t*-BHP	91900	120985	202373	286853	328522	280825	211227	130035	100605
	1.2 mM *t*-BHP	61758	82324	153859	251535	293238	241178	179174	122004	105136
	3.6 mM *t*-BHP	88234	116174	204176	281780	282094	268494	290327	348649	393183
	Luminescence intensity (SD)									
	0 mM *t*-BHP	2156	1748	2249	1691	1132	494	1535	803	1483
	0.2 mM *t*-BHP	3405	2663	5488	8431	6033	3231	2588	2228	2165
	0.6 mM *t*-BHP	2356	3268	7245	9806	7755	4670	4404	3072	2888
Chromosomally integrated ^{P}*TRX2-yNlucCP* reporter assay	1.2 mM *t*-BHP	440	1938	2897	7491	4585	1888	2331	4323	6638
	3.6 mM *t*-BHP	1355	872	1193	2889	2504	2489	2472	3496	2829
	Fold induction (Mean)									
	0.2 mM *t*-BHP	1.70	2.02	3.37	4.60	4.72	4.09	3.39	2.89	2.71
	0.6 mM *t*-BHP	1.33	1.98	3.30	5.13	6.75	6.79	5.56	4.03	3.45
	1.2 mM *t*-BHP	0.90	1.35	2.50	4.50	6.03	5.83	4.72	3.79	3.62
	3.6 mM *t*-BHP	1.28	1.90	3.32	5.04	5.80	6.49	7.65	10.81	13.51
	Fold induction (SD)									
	0.2 mM *t*-BHP	0.02	0.06	0.19	0.21	0.18	0.08	0.21	0.14	0.20
	0.6 mM *t*-BHP	0.02	0.08	0.24	0.26	0.31	0.19	0.23	0.09	0.15
	1.2 mM *t*-BHP	0.02	0.06	0.14	0.24	0.23	0.11	0.17	0.20	0.39
	3.6 mM *t*-BHP	0.06	0.04	0.13	0.10	0.13	0.04	0.30	0.17	0.63

t-BHP conc. (mM)	0	0.2	0.6	1.2	3.6
Relative maximal activity	20.97	78.24	100.00	89.26	85.77

Yeast strains containing two reporter constructs for sensing oxidative stress were cultured with the indicated concentrations of *t*-BHP. Luminescence intensity in each sample was measured at the indicated time intervals. The raw data, including the mean and standard deviation (SD) of luminescence intensity corrected by A_{600} value (measured at time 0), for two reporter assays with or without *t*-BHP are shown for the indicated culture periods (n = 3). Additionally, the mean and SD of fold induction and the relative maximal activity (refer to the legend for S6 Table) in the chromosomally integrated reporter system are shown.
