# Supplementary material for: Sensing chemical-induced genotoxicity and oxidative stress via yeast-based reporter assays using NanoLuc luciferase
Source: PLoS One. 2023 Nov 22;18(11):e0294571. doi: 10.1371/journal.pone.0294571 (PMC10664910; doi:10.1371/journal.pone.0294571)
Supplement: S8 Table — (PDF) [file pone.0294571.s009.pdf]

S8 Table. Raw dataset for Fig 5.

Plasmid-based ^{P}*TRX2-yNlucCP* reporter assay

Culture period (min)	0	10	20	30	40	50	60	80	100	120	140	160	180
Luminescence intensity (Mean)													
0 mM menadione	552611	676212	781566	1067885	1573797	2150807	2626145	3190597	3416607	3466781	3380530	3242480	3070151
0.001 mM menadione	497565	671534	822819	1297319	2045395	2697354	3178109	3623030	3696017	3650038	3535595	3397005	3253282
0.025 mM menadione	509275	848808	1179151	1884342	3170075	4816547	6333167	8551995	9804200	10256334	10223946	9827119	9272823
0.05 mM menadione	787518	1481630	2304035	3361331	4734339	6540629	8674129	12300077	14554087	15824502	16254557	16018477	15460527
0.1 mM menadione	884572	1834319	3107944	4294797	5343893	6162559	6861784	8044189	9034333	9714783	10052104	10046050	9827475
Luminescence intensity (SD)													
0 mM menadione	29935	71374	99450	96905	98415	82625	81006	29326	80954	135349	205123	223887	209241
0.001 mM menadione	33919	32805	12620	104188	142745	230866	308402	420773	396616	414035	367540	306873	224826
0.025 mM menadione	16037	46955	57134	116228	217472	322779	402152	440032	402612	356335	338811	309154	254529
0.05 mM menadione	41630	45312	58383	93542	127511	93037	101941	87207	72457	64147	57433	56416	50297
0.1 mM menadione	28802	22100	33310	41239	39158	46282	37174	3005	9664	16169	11758	15014	17302

Chromosomally integrated ^{P}*TRX2-yNlucCP* reporter assay (Experiment 1)

Culture period (min)	0	10	20	30	40	50	60	80	100	120	140	160	180
Luminescence intensity (Mean)													
0 mM menadione	45091	40126	41508	37808	31411	27181	23871	20800	18657	17396	16084	15004	16661
0.001 mM menadione	64473	91136	168847	203695	209464	191960	169030	143798	128569	117599	109634	99303	92053
0.025 mM menadione	59863	90407	162855	183882	152914	108348	84927	69242	63749	59375	54667	50594	47808
0.05 mM menadione	49252	89485	168247	160092	108076	68151	51908	42477	38034	34456	31621	30171	27959
0.1 mM menadione	49550	53047	57932	53748	44557	37123	32334	27253	24546	23036	21152	19383	18575
Luminescence intensity (SD)													
Time (min)	0	10	20	30	40	50	60	80	100	120	140	160	180
0 mM menadione	392	376	409	1099	734	516	569	823	324	499	213	302	3241
0.001 mM menadione	1603	4184	6739	2054	3364	2999	2793	2598	2192	1747	1220	2229	1873
0.025 mM menadione	1115	5364	7705	8134	9144	7776	8816	6926	6537	5792	3418	2633	2429
0.05 mM menadione	1298	5685	5014	887	1232	1462	559	544	323	1013	666	987	717
0.1 mM menadione	1459	1530	919	1243	1051	436	68	760	123	1052	190	766	188
Fold induction (Mean)													
0.001 mM menadione	1.70	2.02	3.37	4.60	4.72	4.09	3.39	2.89	2.71	2.65	2.71	2.75	2.76
0.025 mM menadione	1.33	1.98	3.30	5.13	6.75	6.79	5.56	4.03	3.45	3.22	3.21	3.22	3.20
0.05 mM menadione	0.90	1.35	2.50	4.50	6.03	5.83	4.72	3.79	3.62	3.71	4.04	4.64	4.87
0.1 mM menadione	1.28	1.90	3.32	5.04	5.80	6.49	7.65	10.81	13.51	15.60	17.19	18.60	18.67
Fold induction (SD)													
0.001 mM menadione	0.02	0.06	0.19	0.21	0.18	0.08	0.21	0.14	0.20	0.24	0.19	0.29	0.34
0.025 mM menadione	0.02	0.08	0.24	0.26	0.31	0.19	0.23	0.09	0.15	0.16	0.23	0.21	0.17
0.05 mM menadione	0.02	0.06	0.14	0.24	0.23	0.11	0.17	0.20	0.39	0.46	0.44	0.64	0.66
0.1 mM menadione	0.06	0.04	0.13	0.10	0.13	0.04	0.30	0.17	0.63	0.79	1.00	1.18	1.20

Menadione conc. (mM)	0	0.001	0.025	0.05	0.1
Relative maximal activity	21.53	27.66	80.32	87.79	100.00

Chromosomally integrated ^{P}*TRX2-yNlucCP* reporter assay (Experiment 2)

Culture period (min)	0	10	20	30	40	50	60	70	80	90
Luminescence intensity (Mean)										
0 mM menadione	161993	223359	230252	234384	245857	248807	248540	235971	232345	222387
0.001 mM menadione	203660	287043	329366	328147	321545	310218	293974	281411	266822	250013
0.025 mM menadione	220712	479274	987844	1242046	1250311	972337	780669	682927	618677	577499
0.05 mM menadione	193689	432374	855710	1160007	1215194	1021767	855577	772606	716868	669439
0.1 mM menadione	165238	337577	634827	816994	931702	918254	853043	791120	729602	700736
Luminescence intensity (SD)										
0 mM menadione	761	6686	10503	12295	13660	15307	14798	14972	14465	11088
0.001 mM menadione	13518	27366	33076	25852	20441	16783	16130	15100	20075	16344
0.025 mM menadione	12338	40919	85513	73285	93442	112795	96171	86101	79273	66455
0.05 mM menadione	7720	26212	44285	25023	17073	17211	24981	28953	32191	33235
0.1 mM menadione	6546	21733	34164	38700	25626	11180	6366	11853	10675	10493
Fold induction (Mean)										
0.001 mM menadione	1.26	1.28	1.43	1.40	1.31	1.25	1.18	1.19	1.15	1.12
0.025 mM menadione	1.36	2.15	4.32	5.33	5.08	3.90	3.13	2.88	2.65	2.59
0.05 mM menadione	1.20	1.94	3.73	4.96	4.96	4.12	3.46	3.28	3.10	3.02
0.1 mM menadione	1.02	1.52	2.77	3.50	3.81	3.71	3.44	3.36	3.15	3.16
Fold induction (SD)										
0.001 mM menadione	0.08	0.10	0.09	0.05	0.07	0.07	0.06	0.02	0.06	0.04
0.025 mM menadione	0.07	0.25	0.55	0.57	0.23	0.21	0.20	0.18	0.17	0.17
0.05 mM menadione	0.05	0.14	0.32	0.29	0.33	0.29	0.24	0.20	0.22	0.19
0.1 mM menadione	0.04	0.14	0.26	0.33	0.30	0.26	0.18	0.16	0.15	0.11

Menadione conc. (mM)	0	0.001	0.025	0.05	0.1
Relative maximal activity	19.90	26.34	100.00	97.19	74.52

Yeast strains containing two reporter constructs for sensing oxidative stress were cultured with the indicated concentrations of menadione. Luminescence intensity in each sample was measured at the indicated time intervals. The raw data, including the mean and standard deviation (SD) of luminescence intensity corrected by A_{600} value (measured at time 0), for two reporter assays with or without menadione are shown for the indicated culture periods (n = 3). Additionally, the mean and SD of fold induction and the relative maximal activity (refer to the legend for S6 Table) in the chromosomally integrated reporter system are shown. Experiments involving the chromosomally integrated reporter were conducted twice.
