# Supplementary material for: Sensing chemical-induced genotoxicity and oxidative stress via yeast-based reporter assays using NanoLuc luciferase
Source: PLoS One. 2023 Nov 22;18(11):e0294571. doi: 10.1371/journal.pone.0294571 (PMC10664910; doi:10.1371/journal.pone.0294571)
Supplement: S9 Table — (PDF) [file pone.0294571.s010.pdf]

S9 Table. Raw dataset for Fig 6.

Culture period (min)	0	10	20	30	40	50	60	70	80	90
Luminescence intensity (Mean)										
0 mM *t*-BHP	84859	127481	146161	200769	292597	288619	208434	151942	120662	100624
0.2 mM *t*-BHP	85274	147368	167916	206364	322175	384723	343151	265999	204175	163877
0.6 mM *t*-BHP	169873	799687	1155109	757212	322674	165417	138958	138673	139951	133760
1.2 mM *t*-BHP	261427	1185791	1488273	705307	316732	198319	177185	165540	161904	155646
3.6 mM *t*-BHP	170425	562410	190833	124279	77356	59309	55107	57275	63295	68700
Luminescence intensity (SD)										
0 mM *t*-BHP	4049	5408	25202	71389	113851	88129	36424	14695	9847	8463
0.2 mM *t*-BHP	3559	5122	3719	8105	26047	32438	21508	11436	5632	5967
0.6 mM *t*-BHP	7875	21121	38310	42290	15675	3626	4731	5094	6724	7414
1.2 mM *t*-BHP	11476	67286	260139	295196	85079	19514	20595	21036	19552	13357
3.6 mM *t*-BHP	7934	38358	16006	9194	5616	3814	2872	2645	4963	1333
Fold induction (Mean)										
0.2 mM *t*-BHP	1.01	1.16	1.19	1.20	1.32	1.46	1.68	1.76	1.70	1.64
0.6 mM *t*-BHP	2.00	6.28	8.13	4.40	1.36	0.65	0.69	0.92	1.17	1.35
1.2 mM *t*-BHP	3.08	9.32	10.25	3.64	1.23	0.75	0.86	1.09	1.34	1.55
3.6 mM *t*-BHP	2.01	4.41	1.33	0.72	0.32	0.23	0.27	0.38	0.52	0.69
Fold induction (SD)										
0.2 mM *t*-BHP	0.07	0.06	0.24	0.49	0.57	0.42	0.22	0.10	0.10	0.09
0.6 mM *t*-BHP	0.08	0.14	1.36	1.80	0.67	0.25	0.16	0.13	0.16	0.20
1.2 mM *t*-BHP	0.07	0.68	1.25	1.05	0.41	0.21	0.08	0.09	0.12	0.09
3.6 mM *t*-BHP	0.02	0.22	0.14	0.28	0.15	0.07	0.04	0.02	0.00	0.05

t-BHP conc. (mM)	0	0.2	0.6	1.2	3.6
Relative maximal activity	19.66	25.85	77.61	100.00	37.79

Culture period (min)	0	10	20	30	40	50	60	70	80	90
Luminescence intensity (Mean)										
0 mM diamide	13654	22915	24800	19902	17320	16705	16620	15104	14963	14848
0.5 mM diamide	17341	30103	35379	30336	25020	23538	22628	21391	20217	20529
1 mM diamide	13573	24466	29400	23420	19877	18898	18436	17147	17247	15833
2 mM diamide	16571	29318	35458	28723	23987	23455	22464	21830	20066	20173
4 mM diamide	9941	18279	21613	18192	15127	14292	13628	13136	12539	12815
Luminescence intensity (SD)										
0 mM diamide	1872	959	1913	1611	2282	2101	1878	1765	1241	2087
0.5 mM diamide	2052	3324	4694	3417	3839	2428	2748	1973	1211	1494
1 mM diamide	1534	2375	3791	3589	2226	2811	2826	2122	2693	1983
2 mM diamide	1142	1241	857	737	1327	2526	1554	1443	1212	1683
4 mM diamide	866	681	1383	1197	1218	290	560	772	564	473
Fold induction (Mean)										
0.5 mM diamide	1.28	1.31	1.43	1.53	1.45	1.42	1.37	1.42	1.36	1.40
1 mM diamide	1.00	1.07	1.18	1.17	1.15	1.13	1.11	1.13	1.15	1.07
2 mM diamide	1.23	1.28	1.44	1.45	1.41	1.43	1.37	1.47	1.35	1.39
4 mM diamide	0.74	0.80	0.88	0.92	0.88	0.87	0.83	0.88	0.84	0.88
Fold induction (SD)										
0.5 mM diamide	0.16	0.13	0.19	0.18	0.20	0.15	0.19	0.08	0.08	0.15
1 mM diamide	0.03	0.06	0.08	0.11	0.05	0.04	0.06	0.02	0.08	0.03
2 mM diamide	0.16	0.05	0.09	0.12	0.20	0.24	0.20	0.20	0.09	0.24
4 mM diamide	0.10	0.05	0.10	0.10	0.10	0.09	0.09	0.07	0.07	0.11

Diamide conc. (mM)	0	0.5	1	2	4
Relative maximal activity	69.94	99.78	82.92	100.00	60.96

Culture period (min)	0	10	20	30	40	50	60	70	80	90
Luminescence intensity (Mean)										
0 saturated zinc oxide	139980	192873	179075	139819	117758	102234	96409	88150	82576	80966
0.125 saturated zinc oxide	118454	158706	161462	119362	100469	88997	80638	76536	73525	67285
0.25 saturated zinc oxide	128993	178840	176553	137232	110953	97963	91269	88056	83300	76798
0.5 saturated zinc oxide	129465	183554	192461	151719	126626	111988	104232	100026	95438	87980
1 saturated zinc oxide	161977	235110	235651	177593	147362	131665	126142	119673	110745	106645
Luminescence intensity (SD)										
0 saturated zinc oxide	2665	7666	7559	11393	5485	5733	3500	2302	2719	3581
0.125 saturated zinc oxide	1659	5974	1968	3669	2244	5241	4770	2588	1016	3542
0.25 saturated zinc oxide	3101	3316	7942	9800	8442	3683	2762	5225	2280	2462
0.5 saturated zinc oxide	3902	10671	7582	8965	5780	4201	5628	4179	3563	2642
1 saturated zinc oxide	3689	5487	11167	16873	13890	13496	9363	9636	9738	9869
Fold induction (Mean)										
0.125 saturated zinc oxide	0.85	0.82	0.90	0.86	0.86	0.87	0.84	0.87	0.89	0.83
0.25 saturated zinc oxide	0.92	0.93	0.99	0.98	0.94	0.96	0.95	1.00	1.01	0.95
0.5 saturated zinc oxide	0.93	0.96	1.08	1.10	1.08	1.10	1.08	1.14	1.16	1.09
1 saturated zinc oxide	1.16	1.22	1.32	1.27	1.25	1.29	1.31	1.36	1.34	1.32
Fold induction (SD)										
0.125 saturated zinc oxide	0.02	0.06	0.05	0.09	0.06	0.08	0.05	0.02	0.02	0.07
0.25 saturated zinc oxide	0.02	0.03	0.05	0.01	0.03	0.02	0.02	0.03	0.03	0.02
0.5 saturated zinc oxide	0.04	0.09	0.07	0.14	0.09	0.10	0.09	0.08	0.08	0.08
1 saturated zinc oxide	0.04	0.02	0.01	0.07	0.09	0.13	0.10	0.10	0.12	0.09

Relative conc. of saturated zinc oxide	0	0.125	0.25	0.5	1
Relative maximal activity	81.85	68.52	75.89	81.67	100.00

Yeast strains containing the chromosomally integrated ^{P}*TRX2-yNlucCP* gene, used for sensing oxidative stress, were cultured with the indicated concentrations of *t*-BHP, diamide, and zinc oxide suspension. The luminescence intensity of each sample was measured at indicated time intervals. The raw data, including the mean and standard deviation (SD) of luminescence intensity corrected by A_{600} value, as well as the fold induction in the reporter assays with or without *t*-BHP, diamide, and saturated zinc oxide, are shown for each indicated culture period (n = 3). Additionally, data for the relative maximal activity are shown. 0 and 1 saturated zinc oxide: samples without zinc oxide suspension and samples with saturated zinc oxide suspension without dilution, respectively. 0.125, 0.25, and 0.5 saturated zinc oxide suspension correspond to 1/8-, 1/4-, and 1/2-diluted saturated suspension, respectively.
